# Effect of Pretreatment with Acids on the N-Functionalization of Carbon Nanofibers Using Melamine

**DOI:** 10.3390/ma15228239

**Published:** 2022-11-20

**Authors:** Tatyana A. Maksimova, Ilya V. Mishakov, Yury I. Bauman, Artem B. Ayupov, Maksim S. Mel’gunov, Aleksey M. Dmitrachkov, Anna V. Nartova, Vladimir O. Stoyanovskii, Aleksey A. Vedyagin

**Affiliations:** 1 Boreskov Institute of Catalysis, 630090 Novosibirsk, Russia; 2 Department of Natural Sciences, Novosibirsk State University, Pirogova Str. 2, 630090 Novosibirsk, Russia

**Keywords:** CCVD, Ni–Cu catalyst, carbon nanofibers, oxidative treatment, N-functionalization, melamine

## Abstract

Nowadays, N-functionalized carbon nanomaterials attract a growing interest. The use of melamine as a functionalizing agent looks prospective from environmental and cost points of view. Moreover, the melamine molecule contains a high amount of nitrogen with an atomic ratio C/N of 1/2. In present work, the initial carbon nanofibers (CNFs) were synthesized via catalytic pyrolysis of ethylene over microdispersed Ni–Cu alloy. The CNF materials were pretreated with 12% hydrochloric acid or with a mixture of concentrated nitric and sulfuric acids, which allowed etching of the metals from the fibers and oxidizing of the fibers’ surface. Finally, the CNFs were N-functionalized via their impregnation with a melamine solution and thermolysis in an inert atmosphere. According to the microscopic data, the initial structure of the CNFs remained the same after the pretreatment and post-functionalization procedures. At the same time, the surface of the N-functionalized CNFs became more defective. The textural properties of the materials were also affected. In the case of the oxidative treatment with a mixture of acids, the highest content of the surface oxygen of 11.8% was registered by X-ray photoelectron spectroscopy. The amount of nitrogen introduced during the post-functionalization of CNFs with melamine increased from 1.4 to 4.3%. Along with this, the surface oxygen concentration diminished to 6.4%.

## 1. Introduction

In recent decades, researchers have paid special attention to the improvement of methods for the introduction of various heteroatoms into the structure of carbon materials (CMs). The growing interest in this research field was due to the fact that heteroatoms introduced into CM structures noticeably change the properties of the CMs and widen the areas of their application [1,2,3]. For instance, nitrogen-containing CMs (N-CMs) are widely used in production of supercapacitors [4,5,6,7,8,9,10,11,12,13] and in catalysis (as catalysts or catalysts’ supports) [14,15,16,17,18,19,20] as well as in biomedicine [21] and even in criminalistics [22].

All the approaches to prepare N-doped CMs can be classified onto two groups: (i) one-pot functionalization, where nitrogen is introduced at the stage of the CM’s synthesis and (ii) post-functionalization, where preliminarily prepared CMs are chemically modified with nitrogen. The one-pot functionalization considers the preparation of N-CMs via a simultaneous pyrolysis or thermal catalytic decomposition of precursors serving as carbon and nitrogen sources [7,8,10,21,23,24,25,26,27,28]. Often, one substrate containing both carbon and nitrogen atoms can be chosen as such a precursor. In the case of post-functionalization, previously prepared (or purchased from a supplier) CMs are treated with some N-containing reagents. This results in bonding of the N-containing functional groups to the CM’s surface or in embedding of nitrogen atoms into the near-surface carbon structure [4,5,6,9,12,14,15,16,17,20,29,30,31,32,33,34,35,36,37]. The following N-containing compounds are usually used for the functionalization of CMs: ammonia [9,12,29,30,34], aniline in a mixture with ammonium persulphate [32], 3-hydroxyaniline [23], acetonitrile [24], glucosamine hydrochloride [31], dicyandiamide [18], melamine [4,6,7,8,9,10,11,12,14,15,16,17,18,19,20,22,23,37], urea [4,9,16,18,21], nitrogen (II) oxide [12], pyridine [23,36], pyrrole [35,38]; tiourea [20], 1,10-phenantroline [36], and ethylene diamine [13,33]. Among them, melamine, due to its high nitrogen content (atomic ratio C/N = 1/2), relative non-toxicity, and cheapness, is more preferable.

On the other hand, carbon nanofibers (CNFs) represent an important class of CMs with unique properties [39]. Therefore, the search for an appropriate method for their controllable synthesis and N-functionalization is an actual direction in modern materials sciences. In the present work, the synthesis of N-functionalized carbon nanofibers (N-CNFs) was performed via the post-functionalization route using melamine as a precursor. The initial CNF samples were prepared by the process of catalytic chemical vapor deposition (CCVD) of ethylene at 550 °C over the microdispersed Ni–Cu alloy (12 wt% Cu) obtained by the method of mechanochemical alloying of metals [40]. The CCVD process starts with a spontaneous disintegration of the bulk Ni–Cu alloy under the action of carbon erosion. This leads to the formation of dispersed active particles catalyzing the growth of the morphologically uniform carbon filaments of submicron diameter [27,40,41,42]. The subsequent acidic treatment of the thus-obtained CNFs results in a complete elimination of mineral impurities such as metallic particles of the catalyst, since no oxide supports (silica, alumina, etc.) were used in the composition of initial catalyst.

Among the diverse and ever-growing areas of practical application, the N-functionalized carbon nanomaterials attract attention in the field of heterogeneous catalysis. N-doped CNTs and CNFs are now considered as prospective substrates for supported metal catalysts. The metallic clusters (or single atoms) can be stabilized in a highly dispersed state by their anchoring at the surface N-sites [14,15,43]. For instance, the catalysts based on N-doped carbon nanomaterials can be effectively used in such processes as hydrodechlorination [36,44,45], selective hydrogenation reactions [46,47,48], formic-acid decomposition to produce hydrogen [49,50], and reversible (de)hydrogenation of liquid organic hydrogen carriers (LOHCs) [51,52,53]. In the latter methods, catalysts assist both to absorb hydrogen during the hydrogenation of unsaturated bonds of a substrate and to release stored hydrogen via catalytic dehydrogenation (reverse reaction). Thus, the development of a simple, versatile, and cost-effective technique for the N-functionalization of CNMs remains of particular interest.

The aim of the present study was to investigate the effect of the preliminary acidic treatment of CNFs on the characteristics of the N-CNF samples obtained by the post-functionalization of the former CNFs with melamine. The CNFs were treated in two regimes: mild washing out from the catalyst using hydrochloric acid and oxidative treatment in a mixture of concentrated sulfuric and nitric acids. The second regime allows not only washing out the catalyst’s particles but oxidizing of the CNF surface as well [4,29,30,35,54,55,56]. The morphology, structure, and chemical composition of the N-functionalized CNF samples were examined by a number of characterization techniques, including electron microscopy, Raman spectroscopy, and X-ray photoelectron spectroscopy.

## 2. Materials and Methods

### 2.1. Chemicals and Materials

Nickel powder (PNK-UT3) and copper powder (PMS-1) used to synthesize the Ni–Cu catalyst were purchased from RusRedMet (Saint-Petersburg, Russia) and NMK-Ural (Yekaterinburg, Russia), respectively. High-purity ethylene (Nizhnekamskneftekhim, Nizhnekamsk, Russia) and high-purity hydrogen (GasProduct, Yekaterinburg, Russia) were used to produce the initial carbon nanofibers. Hydrochloric acid (ultrapure), nitric acid (ultrapure), and sulfuric acid (ultrapure) used for the pretreatment procedures were purchased from SigmaTek LLC (Khimki, Russia). Melamine (Shanxi Fenghe Melamine Co., Yuncheng, China) was used as a nitrogen source for the post-functionalization.

### 2.2. Synthesis of the Catalyst

The Ni–Cu catalyst was prepared by a mechanochemical alloying of metal powders using an Activator-2S planetary mill (Activator LLC, Novosibirsk, Russia), as described elsewhere [40,57]. Initially, nickel and copper powders were premixed in a weight ratio of Ni/Cu = 88/12. Then, 1 wt% of recently prepared CNF was added to the mixture. This was required to accelerate the carbon erosion stage at the beginning of the CCVD process. A specimen of this mixture (10 g) along with grinding balls (340 g; stainless steel; 5 mm in diameter) was loaded into stainless-steel bowls (250 mL in volume). The rotation frequency of the bowls and the platform was controlled using a VF-S15 industrial frequency inverter (Toshiba Schneider Inverter Corp., Nagoya, Japan). To avoid overheating, the bowls were water-cooled. The acceleration of the grinding balls was 780 m/c^2^ (~80 G). The mechanochemical alloying was performed for 5 min. Finally, the bowls were unloaded in air, and the product was separated from the grinding balls and weighed.

### 2.3. Synthesis, Pretreatment and N-Functionalization of CNFs

Initial carbon nanofibers (labeled as cat/CNF, see Table 1) were synthesized via CCVD, as described elsewhere [40]. A specimen of the Ni–Cu catalyst (125 mg) was place into a horizontal quartz tubular reactor installed inside an XD-1200NT high-temperature furnace (Zhengzhou Brother Furnace Co., Zhengzhou, China). The reactor was fed with an argon flow and heated up to 550 °C with a heating rate of 10 °C/min. Then, a reaction mixture composed of ethylene (25 L/h), hydrogen (10 L/h), and argon (20 L/h) was passed through the reactor. The CCVD process was performed at 550 °C for 1 h. The carbon yield was ~110 g_CNF_/g_cat_.

Pretreatment of the cat/CNF sample in hydrochloric acid (labeled as ha-CNF, see Table 1) was carried out by soaking it in an aqueous solution of HCl (200 mL of concentrated HCl mixed with 400 mL of distilled water) for 1 day at room temperature. Then, the sample was washed with distilled water on a glass filter until the pH value of the rinse water was neutral.

Oxidative pretreatment of the cat/CNF samples in a mixture of sulfuric and nitric acids (labeled as ox-CNF, see Table 1) was performed on a water bath at 60 °C for 2 h. The cat/CNF sample was placed into a flask along with 150 mL of distilled water, 100 mL of concentrated HNO_3_, and 100 mL of concentrated H_2_SO_4_. Finally, the sample was washed with distilled water on a glass filter until the pH value of the rinse water was neutral.

Post-functionalization of the ha-CNF and ox-CNF samples with melamine (labeled as N/ha-CNF and N/ox-CNF, respectively, see Table 1) was performed as follows. A specimen (1 g) of the ha-CNF or ox-CNF sample was placed into a cylindrical vessel with 0.476 g of melamine dissolved in 20 mL of distilled water in a water bath at 60 °C. The mixture was maintained for 1.5 h and then dried in a drying oven at 150 °C. The dried sample was placed into a muffle, slowly heated up to 400 °C during 1 h, and calcined at this temperature for another 1 h.

### 2.4. Characterization of the Samples

The N_2_ adsorption/desorption isotherms were recorded at 77 K using an Autosorb-6B-Kr automated adsorption analyzer (Quantachrome Instruments, Boynton Beach, FL, USA). In order to remove the adsorbed moisture and other impurities, before the measurements, a specimen (~150 mg) of each sample was treated in a vacuum (1 Pa) at 250 °C for 20 h. The characteristics of the porous structure were calculated using the ASWin 2.02 software package (Quantachrome Instruments, Boynton Beach, FL, USA). The specific surface area (SSA) values were obtained in accordance with a MA-BET approach described elsewhere [58]. To calculate the outer SSA of the CNF samples, a method of α_S_-curves [59] was applied. An adsorption isotherm for Cabot-BP 280 was used as a reference equation [60]. The pore size distribution was evaluated by a quenched solid density functional theory (QSDFT) method using an integrated model with the following parameters: the adsorbate was nitrogen at 77 K, the pores were slotted, the material was carbon, and the calculation was applied to the adsorption branch of the isotherm.

Scanning electron microscopy (SEM, JEOL, Tokyo, Japan) studies of CNFs were performed using a JSM-6460 electron microscope (JEOL, Tokyo, Japan) at a magnification of 1000–100,000×. The morphology of CNFs was examined by transmission electron microscopy (TEM, Hitachi High-Technologies Corp., Tokyo, Japan) using an Hitachi HT7700 TEM (Hitachi High-Technologies Corp., Tokyo, Japan) working at an acceleration voltage of 100 kV and equipped with a W source.

Raman spectra of CNFs were registered on a Horiba Jobin Yvon LabRAM HR UV-VIS-NIR Evolution Raman spectrometer (Horiba, Kyoto, Japan) equipped with an Olympus BX41 microscope (Olympus Corp., Tokyo, Japan) and a 514.5 nm line Ar-ion laser. To avoid thermal decomposition of the CNF samples, the power of light focused in a spot with a diameter of ~2 μm did not exceed 0.8 mW.

The X-ray photoelectron spectroscopy (XPS) experiments were performed using a SPECS spectrometer (SPECS Surface Nano Analysis GmbH, Berlin, Germany) equipped with a hemispherical PHOIBOS-150-MCD-9 analyzer (SPECS Surface Nano Analysis GmbH, Berlin, Germany). The non-monochromatic MgK_α_ radiation (h*ν* = 1253.6 eV) at 200 W and monochromatic AlK_α_ (h*ν* = 1486.7 eV) at 150 W were used for the primary excitation. The spectrometer was calibrated using the peaks Au4f_7/2_ (84.0 eV) and Cu2p_3/2_ (932.7 eV) attributed to metallic gold and copper foils [61]. The samples were fixed on a holder with 3 M double-sided adhesive copper conducting tape. Peak 4.1 XPS software was applied for spectral analysis and data processing. In order to determine the binding energy values and the areas of XPS peaks, a Shirley background was subtracted. The XPS spectra were fitted with Gaussian–Lorentzian functions for each XPS region. The atomic ratios of elements were calculated from the integral photoelectron peak intensities corrected by the corresponding relative atomic sensitivity factors [61] and the transmission function of the analyzer.

## 3. Results and Discussion

### 3.1. Textural Properties of CNFs

**Figure 1:**
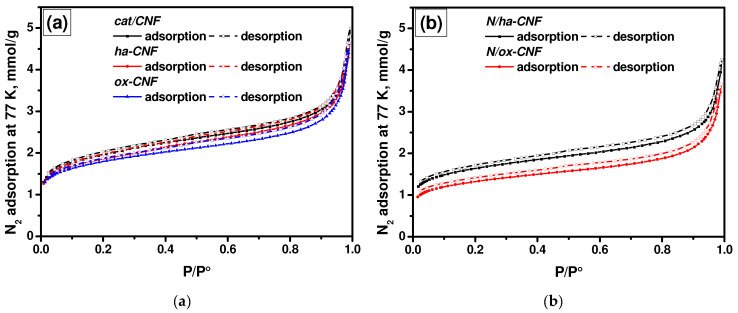
Isotherms of nitrogen adsorption/desorption at 77 K for the studied CNF samples: (**a**) as-prepared and pretreated CNFs and (**b**) N-functionalized CNFs. The samples were treated in a vacuum (1 Pa) at 250 °C for 20 h.

It is well known that any modification or functionalization of the surface of carbon materials should affect their textural properties. To follow such changes, the low-temperature nitrogen adsorption/desorption technique was applied. The obtained adsorption/desorption isotherms for the CNF samples are shown in Figure 1. The shape of the isotherms indicates the presence of large micropores and small mesopores in all studied CNFs. According to IUPAC classification [62], the isotherms are close to Type I(b). They exhibit a narrow hysteresis loop with a shape close to Types H3 or H4. The adsorption and desorption branches do not connect with each other until the relative pressure value reaches 0.025, thus indicating the non-equilibrium character of adsorption on these samples, which can be explained by the swelling of carbon materials. Apparently, the desorption branch is not equilibrium. Therefore, the adsorption branch was used for the further analysis.

The main characteristics of the porous structure of CNFs are summarized in Table 2. As seen from the presented data, the pretreatment and post-functionalization procedures resulted in a decrease in both the SSA and the total pore volume. The average pore diameter, calculated as *4V/A*, where *V* is the total pore volume and *A* is the specific surface area, had changed insignificantly. It should be also mentioned that the effect of the post-functionalization with melamine on the textural characteristics of CNFs was more noticeable than the effect of their pretreatment with acids.

The calculation results for the relative pressure region of 0.5–0.9 of the adsorption branch in comparison with the corresponding data of the QSDFT method are presented in Table 3. As recently reported [63], the comparative method can be used to calculate the SSA of the rough micropores. Comparing the presented characteristics, it can be supposed that the method of α_S_-curves allows the volume of inner pores located inside CNFs to be obtained. Indeed, the values of pore volumes estimated by the α_S_-curves method were close to those calculated by the QSDFT method for the pores less than 2 nm. Therefore, the value of 2 nm seems to be a border value of width for pores located inside and outside the fibers. Almost all the studied samples, except N/ox-CNF, had similar values for the volume of micropores with a diameter < 2 nm to those calculated by the QSDFT method.

**Figure 2:**
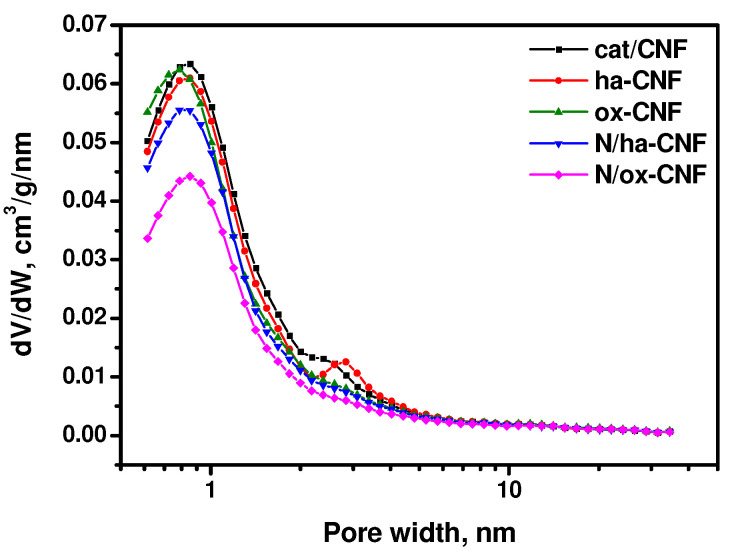
Pore size distribution calculated by the QSDFT method using ASWin 2.02 software.

The pore size distributions calculated by the QSDFT method are shown in Figure 2. As seen, all the samples were characterized by the presence of micropores of ~0.8 nm in width. The samples cat/CNF and ha-CNF also contained small mesopores of 2–3 nm in width. It is evident that the post-functionalization with melamine changed the pore size distribution. Thus, the number of pores of 0.8 nm in size decreased noticeably. It is interesting to note that the pretreatment with hydrochloric acid led to a slight shift of the distribution peak at ~2 nm towards higher sizes without a significant change in its value.

In general, each of modification procedures significantly affected the textural characteristics of CNFs. The main tendencies are a decrease in the contribution of small mesopores and an appearance of macropores with a distribution maximum at ~100 nm.

### 3.2. SEM Characterization of CNFs

**Figure 3:**
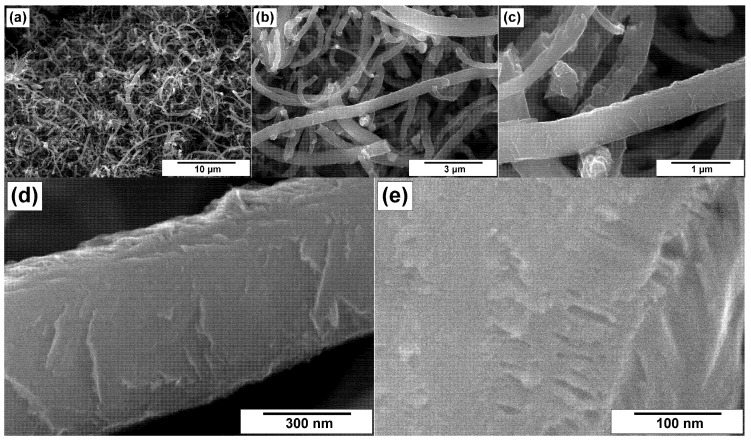
SEM images of the ha-CNF sample at various magnifications: (**a**) 3000×, (**b**) 10,000×, (**c**) 30,000×, (**d**) 60,000×, and (**e**) 100,000×.

**Figure 4:**
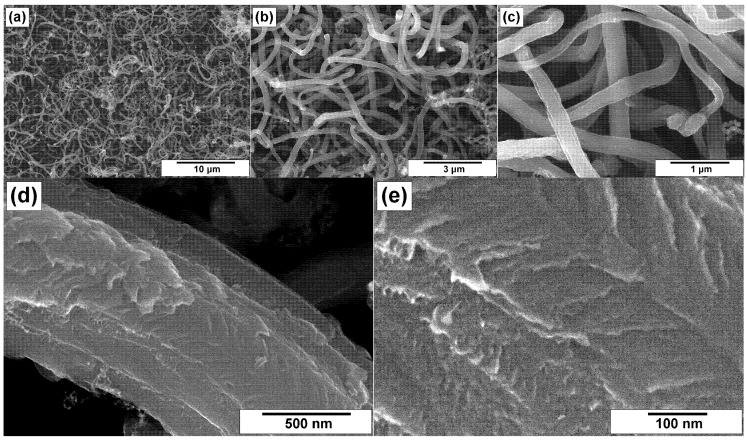
SEM images of the N/ha-CNF sample at various magnifications: (**a**) 3000×, (**b**) 10,000×, (**c**) 30,000×, (**d**) 60,000×, and (**e**) 100,000×.

The modified CNF samples were explored by the SEM technique. Figure 3 and Figure 4 demonstrate the microscopic images for CNFs treated in hydrochloric acid (ha-CNF) and post-functionalized with melamine (N/ha-CNF), respectively. As seen, the ha-CNF sample before the post-functionalization (Figure 3) possessed a fibrous architecture represented by the assemblage of carbon nanofibers. The diameter of the carbon filaments was varied in a range of 100–500 nm. It should be noted that this parameter is defined by the size of the active Ni–Cu particles, catalyzing the growth of CNFs. The images show that all the fibers were of submicron diameter. This is related to the catalyst’s formation process. During the first minutes of interaction with ethylene, the microdispersed Ni–Cu alloy used as a catalyst’s precursor undergoes a rapid disintegration under the action of carbon erosion [40,41,42]. Such a disintegration of the alloy results in the formation of active particles responsible for the growth of CNFs. It is worth noting that the carbon erosion process predetermines, at earlier stages, the formation of a very fluffy carbon product. The carbon filaments within this product do not tangle or form dense agglomerates.

The individual fibers and their fragments are shown in Figure 3d,e. As seen, the fibers are characterized by a relatively dense packing with a few slit-like pores. The mutual orientation of graphene layers defines the following structural types of CNFs: the stacked “pile of plates” structure and the coaxial cones “fishbone” structure [64,65]. The surface of CNF looks rough.

Comparing the ha-CNF samples before (Figure 3) and after the post-functionalization (Figure 4), it can be seen that this procedure (the impregnation with a solution of melamine followed by the calcination at 400 °C) did not practically affect the morphology and the structure of CNFs and did not lead to their destruction.

**Figure 5:**
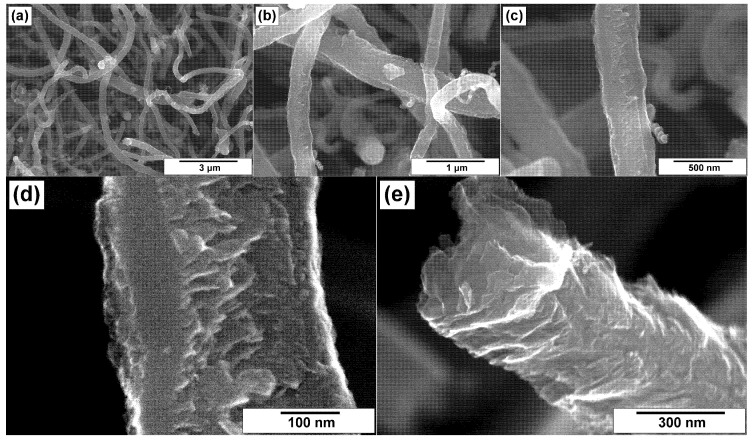
SEM images of the ox-CNF sample at various magnifications: (**a**) 3000×, (**b**) 10,000×, (**c**) 30,000×, (**d**) 60,000×, and (**e**) 100,000×.

On the contrary, pretreatment by heating in a mixture of sulfuric and nitric acids resulted in loosening of the outer surface of CNFs, thus making them more rough (Figure 5c,d). Figure 5e illustrates the packing character of the flakes within the fiber’s body with eliminated metal particle of the catalyst. Nevertheless, the structure integrity of initial CNFs was not broken during such a drastic conditions of the oxidative treatment.

**Figure 6:**
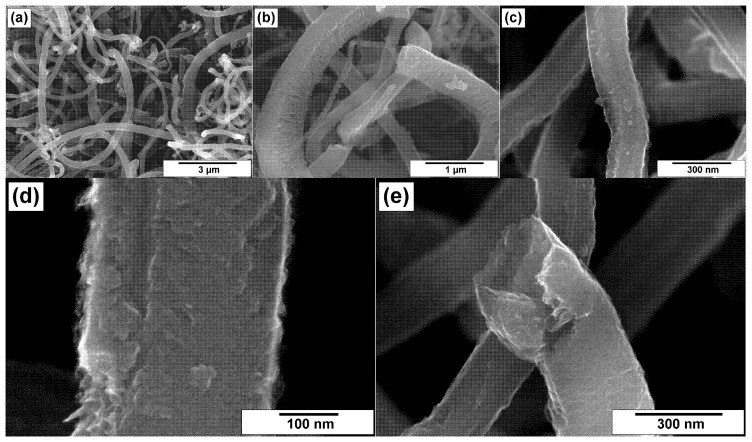
SEM images of the N/ox-CNF sample at various magnifications: (**a**) 3000×, (**b**) 10,000×, (**c**) 30,000×, (**d**) 60,000×, and (**e**) 100,000×.

The influence of the subsequent N-functionalization of the ox-CNF sample on its secondary structure is shown in Figure 6. The fibrous structure of the carbon material remained mainly the same. At the same time, the surface of fibers was maximally loosened (Figure 6c,d). Despite no sufficient changes in the secondary structure being observed, many splits, cleaved facets, and cracks are clearly seen.

### 3.3. TEM Characterization of CNFs

**Figure 7:**
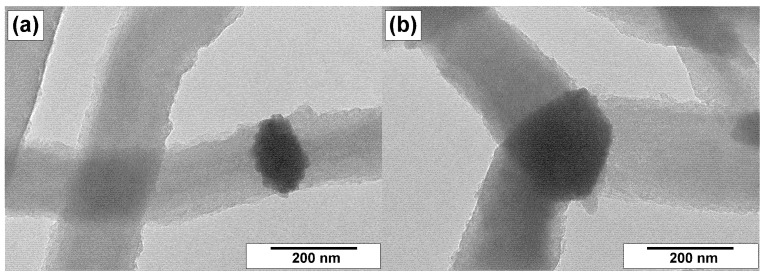
TEM images of the cat/CNF sample prepared via CCVD of ethylene at 550 °C over Ni–Cu alloy: (**a**) two-directional growth of carbon filaments and (**b**) three-directional growth of carbon filaments.

**Figure 8:**
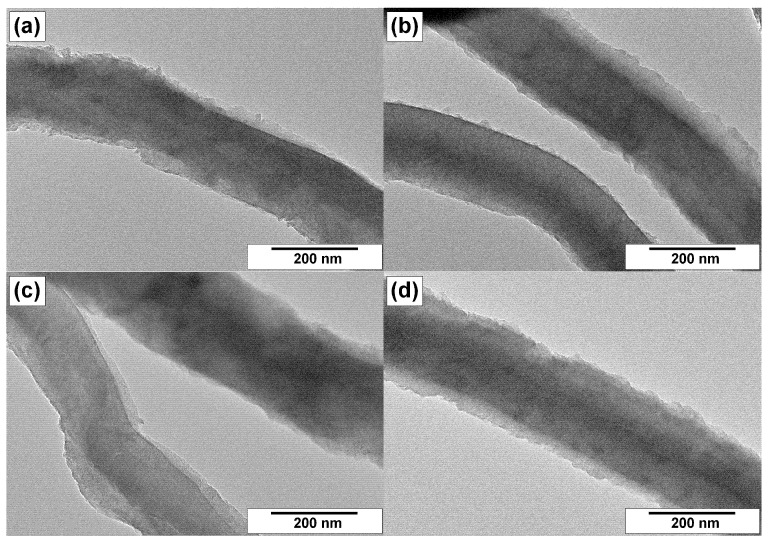
TEM images of the studied samples: (**a**) cat/CNF, (**b**) ox-CNF, (**c**) N/ha-CNF, and (**d**) N/ox-CNF.

**Figure 9:**
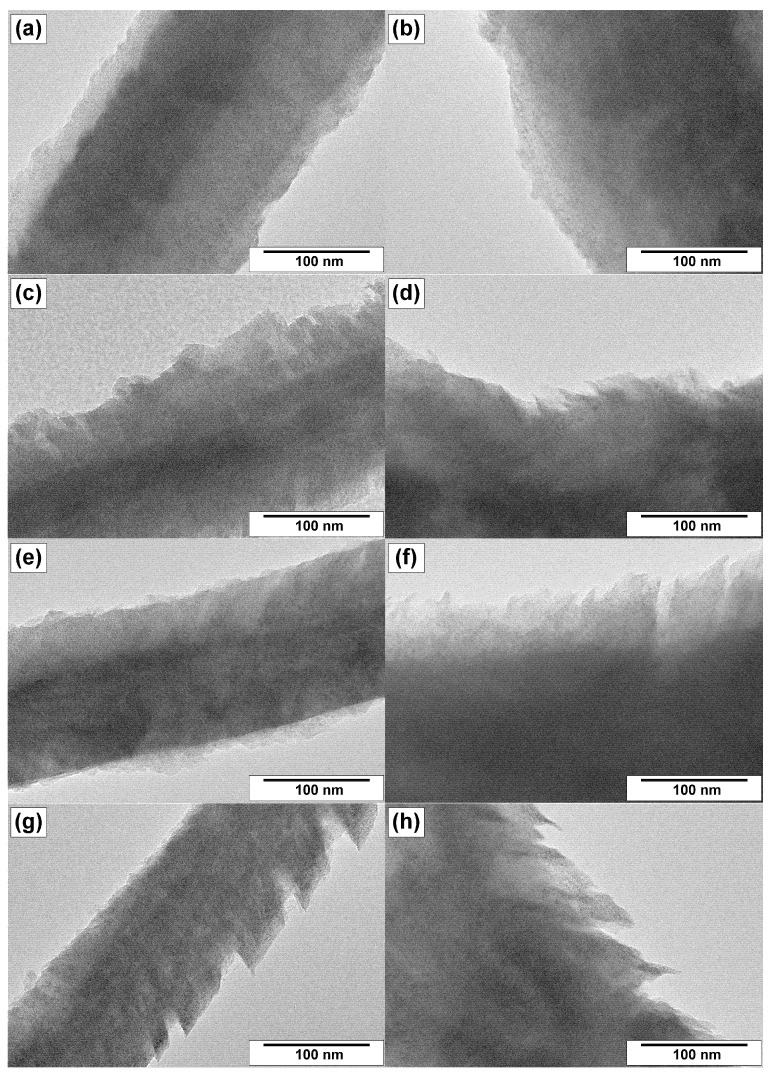
Magnified TEM images of the filament’s surface for the studied samples: (**a**,**b**) cat/CNF, (**c**,**d**) ox-CNF (**e**,**f**) N/ha-CNF, and (**g**,**h**) N/ox-CNF.

Transmission electron microscopy gives more detailed information regarding the morphological and structural features of CNFs. Therefore, the samples were examined by this method as well. The resulting TEM images are collected in Figure 7, Figure 8 and Figure 9.

Figure 7 shows the TEM images for the as-prepared cat/CNF sample obtained via CCVD of ethylene over Ni–Cu alloy. As already mentioned, the growth of carbon filaments occurs as a result of the catalytic action of the dispersed active particles that appear due to the rapid disintegration of the initial Ni–Cu alloy. Such submicron particles of 100–200 nm in size can be clearly seen in Figure 7. One type of particle has a relatively symmetric shape and carries on the growth of carbon filaments in two opposite directions (Figure 7a). At the same time, these particles possess a relief surface without expressed faceting. The second type of particle is alloyed crystallites connected by three or more carbon filaments (Figure 7b). Such particles are clearly faceted. The crystallite plates, where the CNF growth takes place, are perpendicular to the axes of carbon filaments, thus indicating the formation of the stacked structure of CNFs.

It is important to note that in all case, the particles are not entirely covered with carbon. There are places on the surface that are free of carbon, which are responsible for the catalytic decomposition of ethylene molecules and the supply of carbon atoms [66]. These particles are easily accessible for any reagents and, therefore, they can be completely removed from the composition of CNFs even under mild treatment conditions. On the other hand, the acidic pretreatment breaks the integrity of CNFs. As expected, these procedures lead to the breakage of the filaments at the places of removed metal particles and their crushing into two and more pieces. At the same time, the filaments themselves are not fragile. They can stand even boiling in acids without further lengthwise destruction.

Figure 8 and Figure 9 present the TEM images of the same magnification for the samples cat/CNF, ox-CNF, N/ha-CNF, and N/ox-CNF, which allows their structural features to be compared. It is evident that the structure of filaments was not significantly changed during the acidic pretreatment and the subsequent N-functionalization. All the fibers are characterized by a dense packing in the bulk and a loose rough surface.

In some cases, the surface of filaments is covered with a thin layer of carbon with a different character of the graphite packing (Figure 8a,c and Figure 9a,e). The boundary interface is obviously seen in these images. The loose surface of CNFs seems to be more liable for the structural changes under the action of oxidative treatment or N-functionalization. Thus, the carbon fibers with a fringe on the surface were found in the case of CNFs post-functionalized with melamine (Figure 9f,h). At the same time, despite the surface loosening, the structure of the core part of carbon filaments within the composition of N/ha-CNF and N/ox-CNF did not suffer any visual changes.

**Figure 10:**
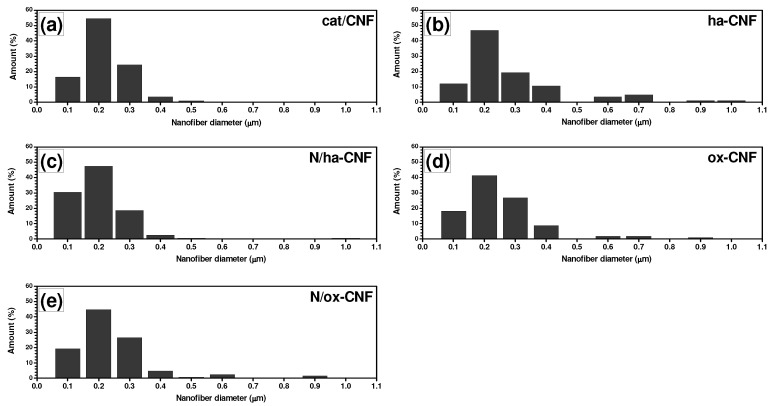
CNF diameter distributions depending on the treatment conditions (based on SEM and TEM data): (**a**) cat/CNF, (**b**) ha-CNF, (**c**) N/ha-CNF, (**d**) ox-CNF, and (**e**) N/ox-CNF.

Based on numerous SEM and TEM data acquired for all studied samples, it was possible to statistically measure the diameter distributions for the carbon nanofibers. The obtained results are presented as diagrams in Figure 10. It is obvious from the comparison of given data that the acidic treatment (both methods) and subsequent N-functionalization had an insignificant effect on the CNF diameter distribution. The measured average diameter of CNFs for all the samples was around 200 nm, regardless to the treatment conditions. The obtained result confirms that the proposed method for N-functionalization of carbon nanofibers appears to be non-destructive.

Therefore, it can be concluded that the proposed approach for the pretreatment of CNFs before their N-functionalization allows the metal particles of the catalysts to be effectively eliminated from the fiber’s structure. The structure and the integrity of the initial filaments as well as their diameter distribution remained almost the same. The post-modification of these CNFs with melamine also did not affect these characteristics noticeably; however, the surface structure was being changed and the surface layer of the filaments became significantly looser.

### 3.4. Raman Spectroscopy Data

**Figure 11:**
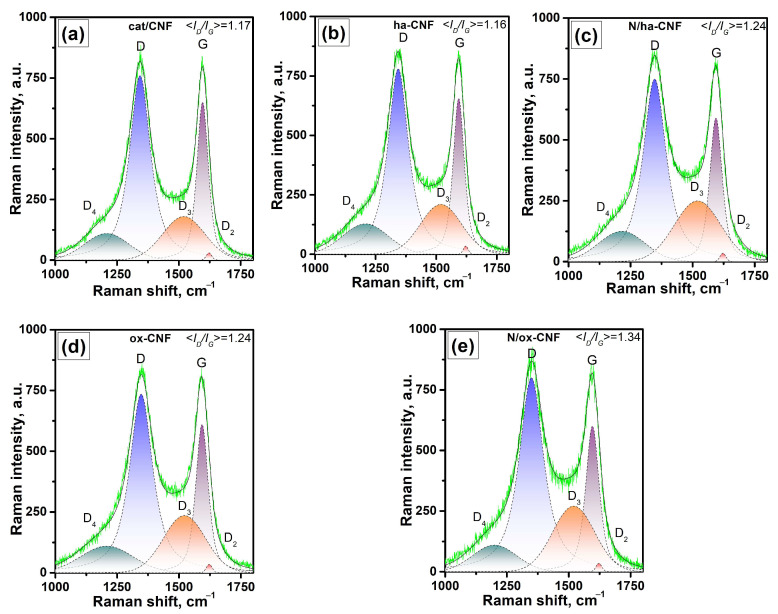
Raman spectra in a region of the first order bands: (**a**) cat/CNF (**b**) ha-CNF, (**c**) N/ha-CNF, (**d**) ox-CNF, and (**e**) N/ox-CNF.

Another informative method to study the structure and orderliness of CNFs is Raman spectroscopy. The typical Raman spectra of the samples in a region of the first order bands are shown in Figure 11. The spectra are characterized by the G bands at ~1595 cm^−1^ corresponding to the allowed vibrations E_2g_ of the hexagonal graphite lattice [67,68] and by the disorder-induced D band (activated A_1g_ mode due to the finite crystal size) at 1345 cm^−1^, which is a characteristic of disordered carbon materials [68,69]. Using a phenomenological three-stage model for the ordering trajectory from tetrahedral amorphous carbon to graphite or the amorphization trajectory considering the change in the G band position for the wavelength of 514.5 nm from 1581 to 1600, 1510, and 1560 cm^−1^, respectively, the studied samples can be assigned closer to nanocrystalline graphite [68].

For all the samples, D_2_ bands can be observed at ~1618 cm^−1^, D_3_ at ~1520 cm^−1^, and D_4_ at ~1208 cm^−1^. The D_2_ bands correspond to a disordered graphitic lattice (surface graphene layers, E_2g_-symmetry) [70], while the two other bands can be assigned to amorphous carbon and disordered graphitic lattice (A_1g_-symmetry) or polyenes [71], which is typical for soot and related carbonaceous materials.

The second order bands are ill-defined and very close to each other in intensity for all the studied samples. The characteristic intensities of the 2D bands at ~2700 cm^−1^ and D + D_2_ at 2934 cm^−1^ were *I_2D_/I_G_* ~ 0.11 and *I_D + D2_/I_G_* ~ 0.14, respectively. Taking into account the half-width values (HWHM) of the G band > 45 cm^−1^ and according to Ferrari and Robertson [68], the cluster diameter (or in-plane correlation length) *L_a_* can be approximated from the *I_D_/I_G_* ratio using the equation *I_D_/I_G_ = C′(λ)·La*^2^, where *C′* is of ~0.0055 for the wavelength of 514.5 nm. The obtained *L_a_* values lie in a range of 13.3–15.7 Å.

**Figure 12:**
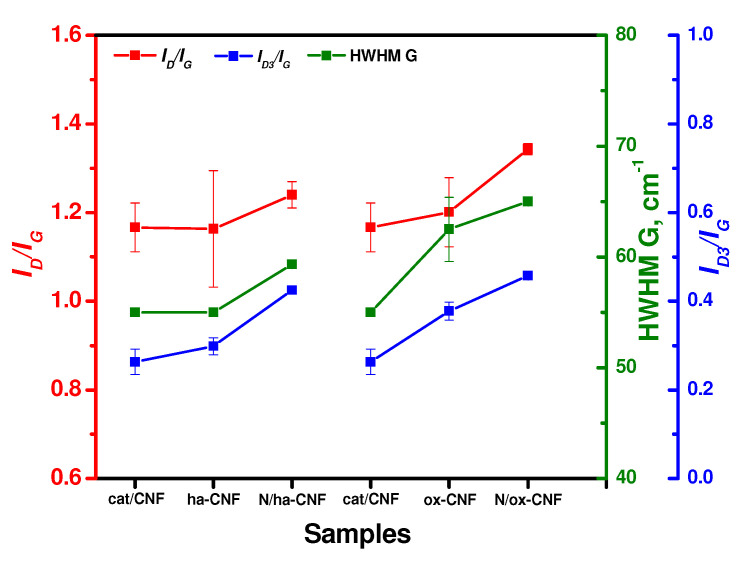
Changes of the *I_D_/I_G_*, HWHM G, and *I_D3_/I_G_* parameters for the studied samples.

The changes of the main parameters (*I_D_/I_G_*, HWHM G, and *I_D3_/I_G_*) resulting from the pretreatment and post-functionalization of CNFs are shown in Figure 12. Note that each value is an average for four points. As seen, the pretreatment of the initial cat/CNF sample in hydrochloric acid led to an insignificant increase in the portion of amorphous carbon (*I_D3_/I_G_*). The oxidative treatment in a mixture of sulfuric and nitric acids (ox-CNF sample), in addition to an increase in the *I_D3_/I_G_* ratio, also increased the HWHM G value and the *I_D_/I_G_* ratio. This indicates a higher number of defects along with an increased portion of crystalline carbon. In other words, this acidic treatment procedure decreased the portion of disordered carbon on the surface of CNFs. In should be noted that a similar effect of etching-out the disordered carbon from the surface of carbon paper using a solution of sulfuric acid being accompanied by an increase in the *I_D_/I_G_* ratio has been reported in the literature [72]. However, the authors interpreted this change in the *I_D_/I_G_* ratio oppositely—as an increase in the disorder degree of carbon, which is the only explanation if no analysis of the HWHM G parameter and no approximation of the *L_a_* value are considered.

The post-functionalization of CNFs with melamine resulted in a synchronous increase in the *I_D_/I_G_* ratio, the HWHM G parameter, and the portion of amorphous carbon *I_D3_/I_G_* (Figure 12). This observation can be interpreted as a rise of the disordered carbon portion on the surface of CNFs. Therefore, it can be concluded that all the modification procedures dealt only with the amorphous carbon and the surface layer of CNFs.

### 3.5. XPS Study of CNFs

**Figure 13:**
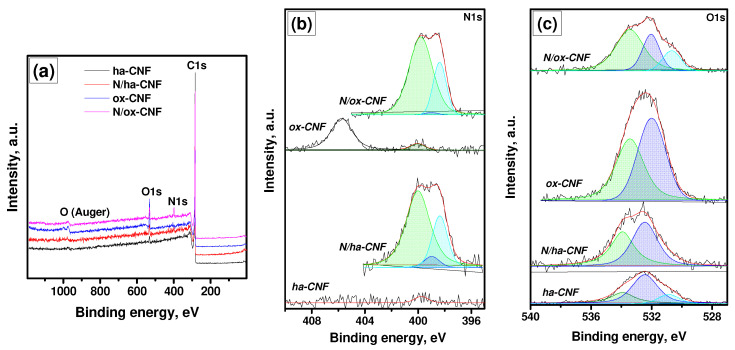
XPS spectra of the studied samples: (**a**) survey spectra, (**b**) N 1*s* region, and (**c**) O 1*s* region.

The qualitative and quantitative estimations of the number of introduced nitrogen and oxygen atoms can be made by means of XPS analysis. According to the survey spectra presented in Figure 13a, the studied CNF samples contain atoms of carbon, oxygen, and nitrogen only. The atomic ratios O/C and N/C are summarized in Table 4.

Figure 13b,c shows the spectra of the N 1*s* and O 1*s* regions recorded using the monochromatic radiation AlK_α_. The binding energy (BE) values, the corresponding species, and their ratios to carbon for all the registered components in these spectra are compared in Table 5 and Table 6. In cases of the ha-CNF and ox-CNF samples, the spectra contain low-intensive peaks at ~ 400 eV, whose appearance could have been caused by the presence of a trace amount of nitrogen. Such a low intensity of these peaks does not allow identifying the state of these species. In addition, in the spectrum of ox-CNF, a state with BE of ~405.8 eV is predominant. This state can be definitely identified as C-NO_2_ groups [73,74]. For both the samples treated with melamine, three states of nitrogen are observed with BE at 398.4, 399.0, and 399.8–400.0 eV. The first (398.4 eV) and second (399.0 eV) states correspond to C-NH_2_ groups [75,76] and C=N-C fragments [75,77], respectively. Note that the state with BE of 398.4 eV can be also attributed to C≡N species [61,75]. The state with BE of ~400 eV is usually assigned to pyrrolic nitrogen [76,77,78]. However, the peak of pyrrolic N is overlapped in XPS spectra by nitrogen of diazine and triazine rings [76].

The presence of an intensive peak at BE near 399 eV in the spectrum of the N/ox-CNF sample allows for the assumption that there was a large amount of residual melamine (or its fragments) in this sample. Note that in the melamine spectrum, nitrogen is in two states with BE of 398.6 eV (C-NH_2_) and 399.2 eV (C=N-C) [75].

The O 1*s* spectra for the samples ha-CNF, N/ha-CNF, and N/ox-CNF contain components that can be attributed to the following groups (Figure 13c, Table 6): C(O)O with BE of 530.7–530.8 eV; C=O with BE of 532.0–532.4 eV; and C-OH with BE of 533.4–534.0 eV [79,80]. In the case of the ox-CNF sample, the components in the XPS spectrum correspond to C=O and C-OH groups, and the latter ones are overlapped with –NO_2_ groups [79,80,81].

Therefore, the performed XPS study confirmed the introduction of heteroatoms (O and N) into the structure of carbon nanofibers during the pretreatment and post-functionalization procedures. Both of the pretreatment regimes caused the complete elimination of metal particles of the catalyst from the composition of CNFs. No components assigned to nickel or copper were observed in XPS spectra. The drastic oxidative treatment (ox-CNF sample) resulted in the modification of the surface of the CNFs with ~11.8 at% of oxygen. Under the mild conditions (ha-CNF sample), the oxidation of the CNF’s surface also took place, but to a lower degree (~2.5 at% of oxygen). The N-functionalization of these samples with melamine allowed for the introduction of ~1.4 and ~4.3 at% of nitrogen for the N/ha-CNF and N/ox-CNF samples, respectively. It is important to note that the oxygen content decreased to 6.5 at% for N/ox-CNF and increased to 4.6 at% for N/ha-CNF. Such an opposite behavior is supposedly connected with the formation of C-NO_2_ groups in the latter case.

By comparing the results of the present research with the literature data related to the synthesis and characterization of the N-doped CNFs, it can be seen that the produced N-CNFs contained a nitrogen amount of several percent which is rather typical for N-CNM materials [4,7,9,12,16,20,29,36]. This is probably related to the comparatively low porosity and flexible structure of CNFs as well as to the application of a post-functionalization method, which mainly affects the surface of carbon fibers. The higher amount of nitrogen can be introduced into the structure of more porous and/or ordered carbons [6,11,14], microspheres [10], and tubes [15], including the use of severe conditions for the functionalization [25].

## 4. Conclusions

In present work, the carbon nanofibers obtained via CCVD of ethylene at 550 °C over an Ni–Cu alloy were modified via the introduction of O and N heteroatoms. The application of a low-temperature nitrogen-adsorption method, SEM and TEM, Raman spectroscopy, and XPS technique gave an opportunity to perform an in-depth study of the modification process. The first stage of this process was the pretreatment with hydrochloric acid (mild conditions) or with a mixture of sulfuric and nitric acids under heating (drastic conditions). Both these procedures allowed the complete elimination of metal particles of the catalysts from the structure of carbon filaments. The elimination of metal particles, in its turn, resulted in a breakage of filaments in places where the particles were located. Thus, the filaments became shorter, and the slight compaction of the carbon material occurred. Therefore, the specific surface area of the samples after the acidic pretreatment diminished insignificantly. According to TEM and Raman spectroscopy data, the modification process dealt mostly with the surface changes. The appearance of oxygen species on the surface of CNFs was registered in both the pretreatment cases. The drastic oxidative conditions gave an oxygen content as high as 11.8 at%. The subsequent post-functionalization with melamine did not practically affect the morphology of the CNF samples. At the same time, it allowed for the introduction of 1.4 and 4.3 at% of nitrogen into the structure of ha-CNF and ox-CNF samples, respectively. Therefore, the proposed approach proved to be applicable for the modification of carbon nanofibers with heteroatoms (O and N), which are primarily demanded as a support for heterogeneous catalysts used in hydrodechlorination, selective hydrogenation, and other processes.

## Figures and Tables

**Table 1:** Designation of the prepared and studied samples.

#	Sample Designation	Description
1	cat/CNF	As-prepared CNFs obtained via CCVD of ethylene at 550 °C over Ni–Cu alloy catalyst
2	ha-CNF	cat/CNF sample pretreated in hydrochloric acid (elimination of metal particles)
3	ox-CNF	cat/CNF sample pretreated in a mixture of sulfur and nitric acids (elimination of metal particles, oxidation of the CNF’s surface)
4	N/ha-CNF	ha-CNF sample post-functionalized with melamine
5	N/ox-CNF	ox-CNF sample post-functionalized with melamine

**Table 2:** The main textural characteristics of CNFs.

Sample	SSA, m^2^/g	Pore Volume, cm^3^/g	Average Pore Diameter (*4V/A*), nm
cat/CNF	161.9	0.173	4.3
ha-CNF	154.3	0.161	4.2
ox-CNF	147.4	0.161	4.4
N/ha-CNF	137.1	0.147	4.3
N/ox-CNF	110.2	0.127	4.6

**Table 3:** The characteristics of micropores in CNFs.

Sample	SSA of the 1st Adsorption layer *, m^2^/g	Volume of Micropores in CNFs *, cm^3^/g	Outer SSA of CNFs *, m^2^/g	Volume of Micropores < 2 nm ^†^, cm^3^/g	Outer SSA of Pores > 2 nm ^†^, m^2^/g
cat/CNF	155.6	0.059	48.5	0.057	20.4
ha-CNF	150.3	0.055	38.5	0.053	20.7
ox-CNF	137.7	0.051	36.4	0.053	17.2
N/ha-CNF	134.5	0.046	34.2	0.048	16.4
N/ox-CNF	109.5	0.035	31.7	0.038	14.1

* calculated by a method of α_S_-curves; ^†^ calculated by a QSDFT method.

**Table 4:** Atomic ratios for the studied CNF samples.

Sample	N/C	O/C
ha-CNF	0.0007	0.025
N/ha-CNF	0.014	0.046
ox-CNF	0.014	0.118
N/ox-CNF	0.043	0.064

**Table 5:** Components of the N 1*s* spectra and their atomic ratios N/C.

BE, eV	Sample				Species	Ref.
	ha-CNF	N/ha-CNF	ox-CNF	N/ox-CNF		
398.4	-	0.0036	-	0.011	C-NH_2_	[75,76]
					C≡N	[61,75]
399.0	-	0.00078	-	0.00048	C=N-C	[75,77]
399.8	-	-	-	0.032	Pyrrolic N	[76,77,78]
400.0	-	0.0098	-	-		
~400.0	0.0007	-	0.0013	-	Not identified	
405.8	-	-	0.013	-	C-NO_2_	[74]

**Table 6:** Components of the O 1*s* spectra and their atomic ratios O/C.

BE, eV	Sample				Species	Ref.
	ha-CNF	N/ha-CNF	ox-CNF	N/ox-CNF		
530.7	-	-	-	0.0095	C(O)O	[79,80,81]
530.8	0.0019	-	-	-		
530.9	-	0.0017	-	-		
532.0	-	-	0.061	0.018	C=O	
532.4	0.019	0.024	-	-		
533.4	-	-	0.057	0.037	C-OH -NO_2_	
533.9	0.0044	-	-	-		
534.0	-	0.02	-	-		

## Data Availability

Data are contained within the article.
